# Socioeconomic variations in chronic obstructive pulmonary disease prevalence, diagnosis, and treatment in rural Southwest China

**DOI:** 10.1186/s12889-020-08687-5

**Published:** 2020-04-19

**Authors:** Le Cai, Xu-Ming Wang, Lu-Ming Fan, Jing-Rong Shen, Ying-Nan Liu, Allison Rabkin Golden

**Affiliations:** 1 School of Public Health, Kunming Medical University, 1168 Yu Hua Street Chun Rong Road, Cheng Gong New City, Kunming, 650500 China; 2 The First Affiliated Hospital of Kunming Medical University, Kunming, China

**Keywords:** Chronic obstructive pulmonary disease, Diagnosis, Treatment, Socioeconomic status, China

## Abstract

**Background:**

Chronic obstructive pulmonary disease (COPD) is a major and growing cause of morbidity and mortality throughout the world. However, there remains a limited understanding of the association between individual socioeconomic status (SES) and COPD diagnosis and treatment worldwide, including in China. This study investigates socioeconomic variations in prevalence, diagnosis, and treatment of COPD in rural China.

**Methods:**

The present study employed a cross-sectional survey design. The study population was composed of Han majority as well as Na Xi and Bai ethnic minority individuals 35 years of age and older living in Yunnan Province from 2017 to 2019. In total, 7534 individuals consented to participate in the study and complete a structured interview as well as a post-bronchodilator spirometry test. Multivariate logistic regression was used to analyze the association between individual socioeconomic status variables and the prevalence, diagnosis, and treatment of COPD.

**Results:**

The age-standardized prevalence of COPD in the present study was 14.3%. Prevalence differed by gender: prevalence for men was 17.1%, versus 11.4% for women (*P* = 0.0001). Overall, levels of diagnosis and treatment of COPD for participants with COPD were 24.2 and 23.1%, respectively. Multivariate logistic regression indicated that higher educational levels and good access to medical services was associated with an overall lower risk of COPD (*P* = 0.032 vs. *P* = 0.018) as well as a higher probability of COPD diagnosis among those with COPD (*P* = 0.0001 vs. *P* = 0.002). Participants with COPD with higher educational levels (*P* = 0.0001) and higher annual household incomes (P = 0.0001) as well as good access to medical services (*P* = 0.016) were more likely to receive COPD medications and treatment than their counterparts. While Na Xi and Bai participants had a higher probability of having COPD (*P* = 0.0001), they had a lower probability of having received a diagnosis or treatment for COPD than Han participants (*P* = 0.0001 vs. *P* = 0.0012).

**Conclusions:**

Future interventions to further control COPD and improve diagnosis and treatment should focus on ethnic minority communities, and those with low education levels, low annual household incomes, and poor access to medical services.

## Background

Chronic obstructive pulmonary disease (COPD) is a major and growing cause of morbidity and mortality throughout the world. The World Health Organization (WHO) predicted a rise in COPD mortality from the fourth leading cause of death in 2004 to the third by 2030, with over 90% of the morbidity and mortality related to COPD occurring in low and middle-income countries [1, 2]. Moreover, COPD prevalence is also increasing globally [3].

In China, COPD ranks among the top three leading causes of death [4]. COPD prevalence has also increased, with the prevalence of COPD among people aged 40 years and over increasing from 2.7% in 1990 to 13.6% in 2015. COPD prevalence in China is also higher in rural areas than urban ones (13.7% vs. 8.6%) [5, 6].

Previous studies established several factors associated with increased risk of COPD. These well documented risk factors include tobacco smoking, secondhand smoke (SHS) exposure, household air pollution (especially exposure to biomass fuels and coal in low and middle-income countries), occupational exposures such as exposures due to tobacco cultivation, history of asthma, and increasing age [7–10]. Moreover, a strong relationship between individual socioeconomic status (SES) and COPD has also been identified, particularly in low and middle-income countries. Regardless of the particular indicators of SES used in the studies, those with low SES were found to have higher prevalence of COPD as well as more respiratory symptoms than those in higher SES groups [11–13]. In accordance with these studies, a negative relationship between SES and prevalence of COPD was also documented in China [14, 15], where those with low educational levels and low household incomes had higher prevalence of COPD.

Despite the major public health challenge COPD presents, COPD remains underdiagnosed and undertreated in countries around the world. In particular, low and middle-income countries have a relatively low rate of COPD diagnosis and treatment compared to high-income countries [11, 15]. Moreover, there remains a limited understanding of the association between individual socioeconomic status and COPD diagnosis and treatment worldwide. A better understanding of the role of individual SES in COPD diagnosis and treatment may inform policy makers designing and implementing appropriate COPD intervention strategies. In China, data on the diagnosis and treatment of COPD are particularly limited in rural and ethnic minority populations, with little data available concerning the associations of SES variables with diagnosis and treatment of COPD.

Thus, this study aims to examine how prevalence, diagnosis, and treatment of COPD differ across socioeconomic factors (including ethnicity, level of education, annual household income, and access to medical services) among the Han majority and two ethnic minority groups (Na Xi and Bai) in rural China. China is a multiethnic nation, with the Han ethnicity accounting for 92% of the population. Located in southwest China, Yunnan Province has the highest number of ethnic minority groups in China, with 25 ethnic minorities in the province. Previous research found a high prevalence of smoking in these populations [16]. However, SES disparities in the development, diagnosis, and treatment of COPD in these populations have not been identified.

## Methods

### Ethical approval

The Kunming Medical University Ethics Committee approved this study prior to commencement of research.

### Study area and population

The present study employed a community-based, cross-sectional, in-person health interview and examination performed from July 2017 to January 2019. From Yunnan Province’s 129 rural counties, Yunnan’s rural population was divided into three geographic strata: valley region, dam region, and mountainous region. One county was then randomly selected from each of these strata, for a total of three selected counties. Subsequently, one Han-populated county, one Na Xi-populated county, and one Bai-populated county were randomly selected. In total, 7800 individuals were recruited to participate using four-stage stratified random sampling to select representative participants 35 years of age and older [17]. Of these, 7534 individuals consented to participate, an overall response rate of 96.6% (3606 men and 3928 women).

### Data collection and measurement

All participants who consented to participate signed informed consent forms and were individually interviewed, face-to-face, by trained interviewers using a pre-tested and structured questionnaire [17]. The interviews covered age, gender, ethnicity, individual SES (level of education, annual household income, and access to medical services), family history of lung disease, self-reported smoking habits, and indoor exposure to biomass fuel. Additionally, information on type of treatment and medications used was obtained from the medical records of participants who reported previous diagnosis of and/or treatment for COPD by a physician. Anthropometric measurements, including height and weight, were taken and post-bronchodilator pulmonary function tests were performed for all participants.

Physicians conducted post-bronchodilator pulmonary function tests with spirometry performed using a portable data logging spirometer (MasterScreen Pneumo, Jaeger, Germany) and tested in accordance with American Thoracic Society guidelines [18]. All participants were led through practice exhalations. Subsequently, with the participants in a sitting position, pre- and post-bronchodilator forced expiratory volume in one second (FEV1) and forced vital capacity (FVC) were measured, and the ratio of these two measurements (FEV1: FVC) was calculated for each subject. If the spirometry was of low quality, the participant was asked to return on another day for an additional spirometry test.

Height and weight measurements were taken using a standardized protocol, in accordance with WHO STEPS methods [19]. Body mass index (BMI) was calculated as weight in kilograms divided by height in meters squared.

### Definitions

COPD was defined as a post-bronchodilator FEV1/FVC < 70% according to the Global Initiative for Chronic Obstructive Lung Disease (GOLD) criteria [20]. Diagnosis of COPD was defined as a participant who self-reported a previous diagnosis of COPD through spirometry testing by a physician, among participants with spirometry-defined COPD. Individuals with COPD who self-reported previously receiving any inhaled short-acting or long-acting bronchodilator or corticosteroid therapy were defined as having received treatment for COPD.

Participants with a BMI < 18.5 kg/m^2^ were defined as underweight. Participants who had smoked at least 100 cigarettes over the course of their lives as well as smoked tobacco products on a daily basis during the survey period were defined as current smokers [21]. Non-smokers who reported exposure to tobacco smoke at home or at work for 15 min or more at least 1 day per week were defined as exposed to SHS [16]. Tobacco cultivators were defined as engaged in tobacco farming within the 12 months prior to the study. Self-reported use of biomass fuel for cooking or heating at home every day over the past 12 months was defined as daily exposure to indoor biomass fuel.

Participants with a walking time of less than 30 min to their nearest village medical center were defined as experiencing good access to medical services while those with a walking time of 30 min or greater were defined as having poor access to medical services. Participants were divided into two educational levels: primary (grade 1–6) or lower, and middle (grade 7–9) or higher. Participants’ annual household income was defined as either low (<$820 US) or high (≥$820 US), with the median value of $820 as the cut-off point.

### Statistical analysis

Descriptive analyses were used to calculate absolute and relative frequencies (%). Counts and percentages were used to present categorical variables. Mean values of FEV1, FVC, FEV1/FVC, and BMI were expressed as mean ± standard deviations ($\overline{X}\pm S$). A chi-squared test was used to compare categorical variables. Age-adjusted prevalence, diagnosis, and treatment of COPD were calculated by directly standardizing to the overall sample. Multivariate logistic regression was used to analyze the association between individual SES variables and the prevalence, diagnosis, and treatment of COPD, adjusted by age, sex, current smoking status, SHS exposure, tobacco cultivator status, daily indoor exposure to biomass fuel, underweight status, and family history of lung disease. Associations were expressed as odds ratios and 95% confidence intervals (CI). All statistical significance calculations were based on a two-tailed *P* value of < 0.05. All data analyses were performed with SPSS 22.0 software.

## Results

Table 1 presents the socioeconomic, lifestyle characteristics, and mean values of anthropometric measurements of the study participants. Of the 7534 total participants, 33.2, 33.6, and 33.2% of participants were of the Han, Na Xi, and Bai ethnicities, respectively. The overall prevalence of current smokers, former smokers, SHS exposure, and family history of lung disease was 30.4, 8.4, 30.3, and 1.2%, respectively. While 9.1% of participants cultivated tobacco, only 3.9% of participants experienced daily exposure to indoor biomass fuel.

**Table 1:** Socioeconomic and lifestyle characteristics and mean value of anthropometric measurements among the study population

Characteristic	n (%) or $$ \overline{X}\pm S $$
Gender	
Male	3606 (47.9)
Female	3928 (52.1)
Age	
35–39 years	285 (3.8)
40–49 years	1484 (19.7)
50–59 years	2001 (26.6)
60–69 years	1872 (24.8)
≥ 70 years	1892 (25.1)
Ethnicity	
Han	2502 (33.2)
Na Xi	2531 (33.6)
Bai	2501 (33.2)
Level of education	
Primary (grade 1–6) or lower	4743 (63.0)
Middle (grade 7–9) or higher	2791 (37.0)
Approximate annual household income	
Good (≥$820)	3738 (49.6)
Poor (<$820)	3796 (50.4)
Access to medical services	
Good	5825 (75.2)
Poor	1874 (24.9)
Current smokers	2291 (30.4)
Former smokers	636 (8.4)
SHS exposure	1588 (30.3)
Tobacco cultivators	689 (9.1)
Daily indoor exposure to biomass fuel	297 (3.9)
Family history of lung disease	89 (1.2)
Spirometry †	
FEV1, L	1.7 ± 1.1
FVC, L	2.1 ± 1.1
FEV1/FVC, %	81.6 ± 13.2
Mean BMI (kg/m^2^, mean ± SD)	23.6 ± 3.6

†pre-bronchodilator, *SHS* secondhand smoke, *BMI* body mass index, *SD* standard deviation *FEV1* forced expiratory volume in one second, *FVC* forced vital capacity

Table 2 shows age-adjusted prevalence, diagnosis, and treatment of COPD by sex, age, and lifestyle factors among the study population. The overall prevalence of spirometry-defined COPD was 14.3% (*n* = 1066), and was higher in men than in women (17.1% vs. 11.4%, *P* < 0.01). The prevalence of diagnosis and treatment of COPD for participants with COPD was 24.2% (*n* = 262) and 23.1% (*n* = 251), respectively. Prevalence, diagnosis, and treatment of COPD increased with age (*P* < 0.01). Men, current smokers, individuals exposed to SHS, and underweight individuals had a higher prevalence of COPD compared to women, non-smokers, non-SHS exposed, and normal weight individuals (*P* < 0.01). Whereas participants who cultivated tobacco and were exposed to indoor biomass fuel daily as well as those who had a family history of lung disease had a higher prevalence of COPD, they also had lower prevalence of COPD diagnosis and treatment than their counterparts (*P* < 0.01).

**Table 2:** Age-standardized prevalence, diagnosis, and treatment of chronic obstructive pulmonary disease by sex, age, and lifestyle factors in Yunnan Province, China

Variable	Prevalence (*N* = 7534)		Diagnosis (*N* = 1066)		Receiving treatment (*N* = 1066)	
	%	95% CI	%	95% CI	%	95% CI
Sex						
Male	17.1	(15.6, 18.1)	24.7	(21.6, 28.5)	23.3	(20.4, 27.1)
Female	11.4**	(10.7, 12.7)	24.5	(20.5, 28.3)	23.8	(19.9, 27.6)
Age						
35–39 years	8.3	(6.8, 10.3)	4.8	(0.7, 9.5)	4.9	(0.8, 9.4)
40–49 years	8.9	(6.0, 12.6)	11.2	(7.3, 18.7)	10.8	(6.7, 17.8)
50–59 years	11.2	(9.7, 12.4)	21.5	(16.9, 27.7)	20.6	(16.1, 26.8)
60–69 years	15.9	(14.1, 17.4)	24.7	(20.3, 30.2)	24.5	(20.3, 30.2)
≥ 70 years	21.3**	(19.5, 23.2)	30.8**	(26.8, 36.8)	28.7**	(24.9, 33.7)
Current smokers						
Yes	19.2	(17.5, 20.3)	18.4	(15.1, 23.2)	17.5	(14.1, 22.0)
No	10.9**	(10.2, 12.1)	27.8**	(24.3, 30.9)	26.7**	(23.4, 29.9)
Former smokers						
Yes	15.6	(14.1, 17.1)	27.6	(23.2, 33.1)	27.1	(22.7, 32.6)
No	13.3	(12.9, 14.8)	26.9	(22.8, 31.7)	26.1	(21.8, 30.2)
SHS exposure						
Yes	20.6	(18.1, 22.0)	28.0	(23.5, 33.4)	26.9	(22.6, 32.4)
No	12.2**	(11.8, 13.5)	26.8	(22.8, 31.5)	26.2	(21.8, 30.4)
Tobacco cultivator						
Yes	17.9	(14.8, 20.4)	22.7	(16.0, 30.8)	20.6	(14.5, 28.9)
No	13.5**	(13.0, 14.7)	24.5	(22.2, 27.7)	23.8	(21.3, 26.7)
Daily indoor exposure to biomass fuel						
Yes	23.7	(18.8, 28.4)	7.1	(3.1, 15.9)	5.6	(2.3, 14.0)
No	13.2**	(12.7, 14.6)	25.7**	(23.2, 28.6)	24.9**	(22.2, 27.5)
Family history of lung disease						
Yes	13.9	(13.2, 14.8)	70.6	(50.8, 85.1)	66.9	(46.7, 80.0)
No	27.3**	(18.9, 37.0)	23.8**	(21.0, 26.2)	22.3**	(20.1, 25.2)
Underweight						
Yes	18.3	(15.4, 23.1)	28.0	(19.4, 39.5)	25.0	(17.1, 36.7)
No	14.2**	(13.1, 14.7)	24.1	(21.7, 27.1)	23.2	(20.9, 26.1)
All	14.3	(13.4, 15.4)	24.2	(22.1, 27.3)	23.1	(22.2, 27.5)

* *p* < 0.05, ** *p* < 0.01

Table 3 indicates the results of multivariate logistic regression analysis for prevalence, diagnosis, and treatment of COPD after adjusting for possible confounding variables. Participants in the Na Xi and Bai ethnic minority groups had a higher probability of having COPD but lower probability of being diagnosed with and receiving treatment for COPD than their Han majority ethnicity counterparts (*P* < 0.01), after adjusting for age, sex, current smoking status, SHS exposure, tobacco cultivation, daily indoor exposure to biomass fuel, underweight status, and family history of lung disease. Among participants with COPD, those with higher educational levels and good access to medical services had increased probability of being diagnosed with COPD (P < 0.01). However, higher educational levels and good access to medical services were associated with a lower risk of COPD (*P* < 0.01). Similarly, COPD patients with higher educational levels, higher annual household income, and good access to medical services were more likely to receive COPD treatment with medications (*P* < 0.05).

**Table 3:** Logistic regression for prevalence, diagnosis, and treatment of chronic obstructive pulmonary disease by socioeconomic status

Variable	Prevalence		Diagnosis		Receiving treatment	
	Adjusted odds ratio^a^	95% CI	Adjusted odds ratio^a^	95% CI	Adjusted odds ratio^a^	95% CI
Ethnicity						
(reference: Han ethnic majority)						
Na Xi ethnic minority	1.83**	(1.34, 2.15)	0.17**	(0.11, 0.27)	0.17**	(0.11, 0.28)
Bai ethnic minority	1.42**	(1.20, 1.68)	0.29**	(0.20, 0.42)	0.28**	(0.19, 0.41)
Level of education						
(reference: Primary (grade 1–6) or lower)						
Middle (grade 7–9) or higher	0.89*	(0.75. 0.99)	2.85**	(2.00, 4.06)	2.79**	(1.95, 3.99)
Approximate annual household income						
(reference: Poor)						
Good	1.02	(0.89, 1.17)	1.13	(0.83, 1.56)	1.16*	(1.04, 1.60)
Access to medical services						
(reference: Poor)						
Good	0.85*	(0.73, 0.99)	1.33**	(1.18, 1.56)	1.14*	(1.02, 1.58)

* *p* < 0.05, ** *p* < 0.01, ^a^ adjusted for age, sex, current smokers, SHS exposure, tobacco cultivation, daily indoor exposure to biomass fuel, underweight status, and family history of lung disease

## Discussion

The findings indicate individual SES has both positive and negative associations with prevalence, diagnosis, and treatment of COPD in the rural southwest Chinese adult population. Moreover, prevalence, diagnosis, and treatment of COPD varied by ethnicity, with minority groups experiencing higher prevalence of COPD but lower probability of being diagnosed with and receiving treatment for COPD than their Han counterparts with COPD.

The present study also demonstrates a markedly higher prevalence of spirometry-defined COPD among the surveyed population relative to the rest of China, underscoring COPD’s high prevalence in rural southwest China: the overall prevalence of COPD was greater than the prevalence observed in two national COPD prevalence studies (8.6 and 13.6%) [6, 14], studies in other regions of China (9.8%) [10], as well as results from the global and regional estimates of COPD (11.7%) [3]. Moreover, the prevalence of COPD among the studied population was higher in men than in women, and increased with age. This finding aligns with many previous studies conducted in both high-income and low and middle-income countries [3, 4, 6, 11, 12]. The much higher prevalence of COPD in the present study may possibly result from the higher prevalence of current smoking (62.1% among males) in the participant population, as smoking plays a critical role in lung disease development [22]. The results suggest that the regions under study are in need of further interventions to prevent and control COPD. However, the prevalence of COPD diagnosis and treatment among participants with COPD (24.2 and 23.1%) in the present study was higher than previous Chinese studies (23.6 and 10%) [14, 23], indicating progress in improving access to healthcare in rural China.

Tobacco smoking, SHS exposure, daily indoor exposure to biomass fuel, underweight status, and family history of lung disease have been well documented as central risk factors for COPD among low and middle-income countries [7–11]. Our results are consistent with these studies, as current smoking status, exposure to SHS, daily indoor exposure to biomass fuel, underweight status, and family history of lung disease were positively associated with the probability of having COPD. In addition, occupation was also an important contributor to the development of COPD in the present study as participants who cultivated tobacco experienced higher prevalence of COPD. This is possibly due to tobacco farmers’ higher prevalence of current smoking than non-tobacco farmers (37.0% vs. 29.7%) in the surveyed population, as found in a previous study in China [16]. However, among those with COPD, current smokers and participants exposed to daily indoor biomass fuel had lower prevalence of diagnosis and treatment than their counterparts, indicating implementation of diagnostic testing and administering medications for COPD should be given special emphasis in COPD patients who smoke tobacco and/or are exposed to indoor air pollution.

This study indicates ethnic differences in COPD prevalence, diagnosis, and treatment in the study region. Specifically, Na Xi and Bai ethnic minority participants had higher prevalence of COPD than Han majority participants, whereas among those with COPD, Na Xi and Bai ethnic minority participants had lower prevalence of COPD diagnosis and treatment than the Han majority ethnicity. The higher prevalence of COPD among ethnic minority groups possibly results from the fact that Na Xi and Bai ethnic minority participants had a higher prevalence of male current smokers (63.2 and 65.3%) as well as a higher rate of tobacco cultivators (18.6 and 6.4%) compared to Han majority participants (57.2% vs. 3.7%). Moreover, the lower prevalence of COPD diagnosis and treatment among ethnic minority patients may be attributed to ethnic minority groups’ economically disadvantaged status in China, and the fact that access to diagnostic testing and effective COPD medications are often more limited in economically depressed populations [24]. These ethnic differences in COPD prevalence, diagnosis, and treatment indicate ethnicity is a crucial factor in COPD, and this presents a challenge for local governments to take particular efforts to further control COPD for all, while improving access to healthcare in minority communities.

The present study indicated low educational attainment and poor access to medical services are associated with higher odds of having COPD but a lower probability of COPD diagnosis and treatment. This inverse relationship between level of education and prevalence of COPD has been found in many previous studies [12–15, 24]. Education may influence health by improving one’s ability to utilize health services and seek effective treatment. Our findings suggest that access to healthcare is a key determinant in prevention, diagnosis, and treatment levels of COPD. Further, they highlight an urgent need for improving community-based COPD prevention strategies as well as increasing diagnosis and treatment measures focused particularly on individuals with low educational attainment.

There is growing evidence in the literature that those with low income are more likely to develop COPD [11, 12, 25]. However, the present study did not find this to be true in the surveyed rural communities. This finding did however align with two previous studies in China, the China Pulmonary Health (CPH) study [6] and a chronic disease risk factor surveillance study [15] that found low household income was associated with high prevalence of COPD in urban areas but not rural ones. The reasons behind this discrepancy are currently unclear. However, while the present study yielded no evidence supporting any association between individual household income and COPD, it did find that those with higher incomes are more likely to be treated for their COPD with medications. This possibly results from the fact that higher incomes afford greater ability to obtain needed health services and medications. The present study’s findings thus underscore an urgent need to improve access to healthcare services and medications for those with low-incomes.

The large sample size and high response rate in the present research is a fundamental strength of this study. Yet the present results are limited in three ways. First, this study was limited by its cross-sectional design; causal relationships cannot be determined. Second, since some individuals with COPD may not require treatment because they are asymptomatic, this study may have underestimated the treatment of COPD. Third, the present findings were based on a random sampling of three ethnic groups, limiting their generalizability.

## Conclusions

In conclusion, the present study indicates spirometry-defined COPD is highly prevalent in rural China in both Han majority and ethnic minority communities. The results suggest that future COPD control efforts and the implementation of diagnostic and treatment guidelines in COPD focus on minority, low-income, less educated populations with poor access to medical services.

## Data Availability

The datasets used and/or analyzed during the current study is available from the corresponding author on reasonable request.

## Abbreviations

BMI: Body mass index
CI: Confidence interval
COPD: Chronic obstructive pulmonary disease
FEV1: Pre- and post-bronchodilator forced expiratory volume in one second
FVC: Forced vital capacity
GOLD: Chronic obstructive lung disease
WHO: World Health Organization
